# Acupressure

**Published:** 1860-05

**Authors:** 


ACUPRESSURE.—This novel process for arresting hemorrhage in surgical operations appears to be steadily gaining favor with our British brethren. We have not ourselves had an opportunity of trying it, but shall certainly do so at the first favorable moment. We republish, in another part of the REVIEW, an interesting paper upon the subject, which we have just received from our distinguished confrere, Professor Simpson, the inventor of the operation.

